# Characterization of Mechanical Oscillations in Bismuth Selenide Nanowires at Low Temperatures

**DOI:** 10.3390/mi14101910

**Published:** 2023-10-07

**Authors:** Liga Jasulaneca, Raimonds Poplausks, Juris Prikulis, Elza Dzene, Tom Yager, Donats Erts

**Affiliations:** 1 Institute of Chemical Physics, University of Latvia, 19 Raina Blvd., LV-1586 Riga, Latvia; raimonds.poplausks@lu.lv (R.P.); juris.prikulis@lu.lv (J.P.); elza.dzene@lu.lv (E.D.); donats.erts@lu.lv (D.E.); 2 Institute of Solid State Physics, University of Latvia, 8 Kengaraga Str., LV-1063 Riga, Latvia; tom.yager@cfi.lu.lv; 3 Faculty of Chemistry, University of Latvia, 1 Jelgavas Str., LV-1004 Riga, Latvia

**Keywords:** resonance detection, radio-frequency, 1D nanomaterials, bismuth selenide

## Abstract

A single transistor preamplifier circuit was designed to facilitate electrical detection of mechanical oscillations in nanoelectromechanical systems (NEMSs) at low temperatures. The amplifier was integrated in the close vicinity of the nanowire inside the cryostat to minimize cabling load and interference. The function of the circuit was impedance conversion for current flow measurements in NEMSs with a high internal resistance. The circuit was tested to operate at temperatures as low as 5 K and demonstrated the ability to detect oscillations in double-clamped bismuth selenide nanowires upon excitation by a 0.1 MHz–10 MHz AC signal applied to a mechanically separated gate electrode. A strong resonance frequency dependency on temperature was observed. A relatively weak shift in the oscillation amplitude and resonance frequency was measured when a DC bias voltage was applied to the gate electrode at a constant temperature.

## 1. Introduction

Nanoelectromechanical systems (NEMSs) are a continuously developing technology that exploit the quasi-static and dynamic mechanical motion of nanostructures to convey signals with high energy efficiency [1,2,3]. In the quasi-static mode, structural deformations of the nanostructure are used to switch from one stability point to another, also usually employing formation of electrical and mechanical contact. This is used in logic and memory elements (switches and relays) to create fast switching suitable for harsh environment applications [2,3,4]. Dynamic NEMSs exploit their vibrations in non-contact mode. They are characterized by high resonance frequencies and a broad tuning range [5,6], enabling precise and versatile devices such as high-performance resonators, filters, oscillators and sensors [7,8]. Besides device applications, mechanical phenomena can also be exploited as a means for material property characterization of nanoscale components (e.g., effective Young’s modulus [9,10]) for studying energy losses, current-induced processes [6,11] and even sensitive mesoscopic interference phenomena [12].

There are various transduction methods that can be used to excite and detect mechanical resonances in nanomaterials, including electric [13,14], electrothermal [6], piezoelectric [15] and optical [6,15] methods. Due to the extremely small displacement amplitudes of the resonant structures in the range of nanometers or less, detection of the vibrations is the most complicated part of the experiment. Detection schemes widely applied in microelectromechanical systems (MEMSs) need to be adapted for nanoscale use. The detection often needs to be performed in customized setups, e.g., to conduct experiments at very low or high temperatures, in a vacuum or in high magnetic fields, requiring increasing amounts of wiring. Low temperature applications have been actively explored for quantum computers [16] and space technologies.

Among the readout schemes, direct observation with electron and light microscopes [17,18] and electrical [13,14] and optical [6,15] schemes are commonly employed. Electrical detection schemes are widely used for resonance registration, as they are easy to integrate with other components and, in the case of one-dimensional (1D) materials, have larger coupling than, e.g., optical detection schemes. Although optical detection has been adapted to detect resonance at low temperatures [19], its implementation is more complicated [20]. Optical probing may induce local heating that may change the properties of sensitive nanomaterials [15]. By using the same electrical setup in a cryostat both for magnetotransport measurements and resonance detection, it would be possible to obtain information about charge carriers and mechanical properties simultaneously without added complexity. A common electrical detection technique employs electrical mixing [21,22] that allows for efficient detection of the resonance by converting it to another frequency that does not suffer from parasitic elements of the circuit, such as electrodes and wires contacting the nanostructure. Another approach is to employ a local gate electrode close to the vibrating nanostructure [13,23] to minimize parasitic circuit contribution and facilitate readout at the same frequency as the excitation, thus increasing the detection speed.

Bismuth selenide (Bi_2_Se_3_) nanostructures are attractive for various applications, for example, spintronic and energy-efficient electronic devices [24,25], owing to their topological insulator properties and the possibility to access and manipulate unique surface states [26]. Another application is their potential use as NEM switch-active elements at low temperatures [27]. Resonant frequency detection allows rapid characterization of NEM switching elements and presents the possibility for on-chip voltage reduction [28] of a NEM switch operating at low temperatures. In addition, as a semiconductor, Bi_2_Se_3_ exhibits gate tunability that would allow modulation of ON-state current in the NEM switch and to employ direct readouts of the resonance frequency, similarly to graphene [13,29].

In this study, we develop a setup for the electrical detection of the mechanical resonance of nanostructures at low temperatures by integrating a preamplifier next to the measured device. We show that this setup allows efficient detection of the resonant frequency of 1D nanostructures by measuring individual double-clamped Bi_2_Se_3_ nanowires. The detection setup is generic and may potentially be used for studies of other NEMSs.

## 2. Materials and Methods

### 2.1. Device Fabrication

Devices with suspended Bi_2_Se_3_ nanoribbons on Si substrates were fabricated using electron beam lithography (EBL, eLINE Plus, Raith, Dortmund, Germany) on commercially available high-resistivity Si substrates (10 kΩ cm) with a 200 nm-thick dry thermal SiO_2_ layer. Trenches for gate electrodes were patterned using reactive ion etching (PlasmaPro 100 Cobra ICP RIE, Oxford Instruments, Abingdon, UK) with SiO_2_ using a PMMA A9 electron beam resist as a mask and CHF_3_-O_2_ (20 sccm CHF_3_ and 5 sccm O_2_ at 10 °C and 200 W HF power) gas as the etchant. Trenches with depths down to 270 nm were fabricated, followed by another lithography step for thermal evaporation (Sidrabe Vacuum, Riga, Latvia) of a 5 nm/60 nm Ti/Au metal layer in the trenches to form local gate electrodes.

Bi_2_Se_3_ nanoribbons were grown using a high-yield vapor–solid synthesis method with a Au catalyst reported previously [30]. They were transferred mechanically to the as-fabricated substrate with gate electrodes. Suitable nanoribbons were identified using an optical microscope for patterning source and drain electrodes via electron beam lithography. To create ohmic contacts between Bi_2_Se_3_ and Au, native oxide covering Bi_2_Se_3_ was etched in a hydrochloric acid/acetic-acid-based solution prior to metallization with 200 nm Ti/Au layers.

The final devices were inspected via scanning electron microscopy (SEM, Hitachi FE-SEM S-4800, Hitachi, Chiyoda City, Tokyo, Japan) to check that they had not adhered to the gate electrode and there was a continuous metal layer over the nanostructure. The thickness of the nanoribbons and depth of the trenches were measured using atomic force microscopy (AFM, Asylum Research MFP-3D, Santa Barbara, CA, USA).

Nanowires with thicknesses from 35 to 224 nm, widths from 124 to 266 nm and lengths from 7.6 to 8.3 μm, with a distance to the gate electrode of 157 nm and two-terminal resistances up to 50 kΩ, were analyzed using an electrical detection method described below.

### 2.2. Electronic Measurements

**Figure 1:**
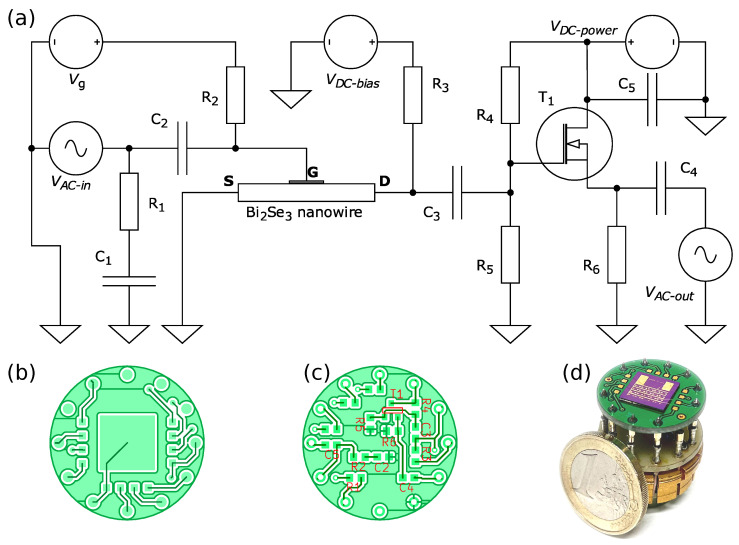
(**a**) Schematic of the preamplifier circuit. Element values: R_1_—50 Ω; R_2_, R_4_, R_5_—2 MΩ; R_3_ equal to DC resistance of Bi_2_Se_3_ nanowire; R_6_—1.2 kΩ; C_1_, C_2_, C_3_, C_4_—0.1 µF; C_5_—0.5 µF; T_1_—2N7002. Layout of the PCB shown from (**b**) top and (**c**) bottom sides. (**d**) PCB mounted on a PPMS holder with the sample bonded to the top.

Figure 1 shows the diagram of the preamplifier circuit inside the physical property measurement system (PPMS, Dynacool 9T, Quantum Design, San Diego, CA, USA). Bi_2_Se_3_ nanowires and the resistor R_3_ formed a voltage divider, which was connected to a $V_{DC-bias}$ source (Keithley-2400, Tektronix, Bracknell, UK). Current flow measurements at the $V_{DC-bias}$ source indicated whether the Bi_2_Se_3_ nanowire was damaged, while R_3_ also served as a current limiter. The capacitor C_3_ connected the AC component of the voltage drop across the Bi_2_Se_3_ nanowire to the gate of the transistor T_1_ (2N7002, Diodes Incorporated, Plano, TX, USA), which operated as a source follower (SF). The advantage of SF circuits (common drain amplifier) is that they are relatively immune to transistor parameters and other component value variations at extreme temperatures. The resistors R_4_ and R_5_ ensured that the transistor gate was biased above the gate-source threshold voltage $V_{T}$ of T_1_. For an ideal SF scheme, the AC component of the voltage drop across the resistor R_6_ is equal to that of the Bi_2_Se_3_ nanowire, but with a much lower impedance for driving the load at terminal $V_{AC-out}$ via capacitor C_4_. The resistance of R_6_ was chosen relatively high to avoid heating inside the cryostat. C_5_ is a bypass capacitor for the supply voltage $V_{DC-power}$. The conductivity of the Bi_2_Se_3_ nanowire was modulated by the middle (G) electrode, which through the capacitor C_2_ was connected to the input terminal $V_{AC-in}$ and through the resistor R_2_ was biased by a DC voltage $V_{g}$ that was provided by a programmable external source (Keithley-6430, Tektronix, Bracknell, UK). Monitoring the DC current at $V_{g}$ indicated whether a jump-to-contact event had occurred. To avoid the risk of nanowires contacting the gate electrode, the gate voltage was kept in the range of ±8 V. The R_1_C_1_ matching network additionally provided a load of $V_{AC-in}$ voltage for diagnostic of electric connections.

All components including the Si substrate with the Bi_2_Se_3_ nanowire were assembled on a miniature printed circuit board (PCB) (Figure 1b–d), that was mounted on a sample holder of the PPMS. The connections between the PCB and sample substrate were made with a 25 μm diameter Au bonding wire using a UniTemp WB-100 (UniTemp, Pfaffenhofen, Germany) ultrasonic wire bonder (Figure 1d). The holder with the bonded sample was loaded into the PPMS and measurements were taken at a pressure of approximately 0.6 Torr.

A $V_{AC-in}$ signal was generated using a vector network analyzer (VNA, Rohde & Schwarz ZNB 8, Rohde & Schwarz, Columbia, MD, USA) port 1. The output signal $V_{AC-out}$ was connected to VNA port 2 and the S_21_ parameter was used to characterize the system amplitude transfer function A(f). The typical operating parameters were $V_{AC-in,rms}$ 0.22 V, $V_{DC-power}$ 15 V and $V_{DC-bias}$ 0.04 V with a corresponding $I_{SD}$ in the range of 0.5–1.0 μA.

According to the manufacturer’s datasheets, the operating temperature of the electronic components is specified to a limited range, typically above −55 °C. However, they often can function even at cryogenic temperatures [31,32], although with deviations from guaranteed parameter tolerances. Indeed, at low temperatures, the resistor values were several times higher than nominal, as measured by the PPMS (not shown). The cabling in the PPMS is designed for DC or low frequency measurements and at higher frequencies suffers from parasitic reactive elements, interference and poor impedance matching. Moreover, no proper VNA calibration was possible for samples inside the cryostat. To account for these issues, the response of the electrical circuit was characterized prior to the nanowire measurements by using:

1. A standard MOSFET as a test three-terminal device to determine if the amplification of the circuit remains consistent at low temperatures;
2. A 5 MHz quartz resonator to examine gain and frequency variations in response to lowering the temperature;
3. A standard 20 kΩ resistor to check for unwanted resonances in the circuit.

These tests allowed determination of the operation bandwidth. Here, we observed that amplification of the signal remains consistent until 5 K, which is the lowest temperature achievable in our setup, and unwanted resonances do not appear in the frequency range from 0.1 MHz to 10 MHz.

The 5 MHz quartz resonator (Abracon ABLS-5.000MHZ-B2-T, Abracon, Spicewood, TX, USA) was connected to G and D terminals (Figure 1) with V_*DC−bias*_ disconnected. A standard 20 kΩ resistor was connected to S and D terminals.

## 3. Results and Discussion

**Figure 2:**
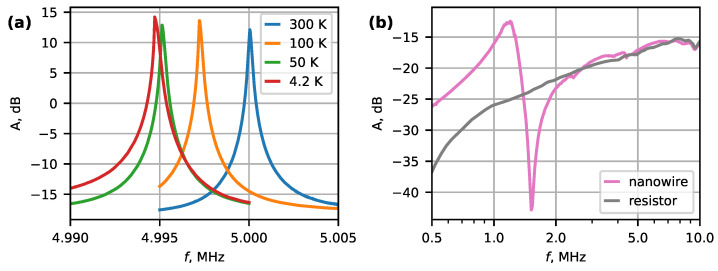
(**a**) Amplitude spectra of a 5 MHz quartz resonator at different temperatures. (**b**) Amplitude spectra of a Bi_2_Se_3_ nanowire device (No. 1) and a 20 kΩ resistor at 8 K.

Figure 2a shows the amplitude spectra for a 5 MHz quartz resonator in temperature range of 300 K to 4.2 K. There is a small ∼0.005 MHz frequency shift in the resonance, as the lower operating temperature limit of quartz resonator is −20 °C. However, lowering the temperature does not impact amplification, as the gain of the system remained nearly constant with only a ∼2 dB variation until approximately 4 K. The general characteristics of MOSFET operation at these temperatures are expected to change insignificantly [32]. Although changes in DC output due to variation in the gate threshold voltage with temperature could be expected, by operating the circuit as a source follower, the AC component is insensitive to parameter changes.

Although an increase in the quality factor of the quartz resonator alone would be expected by lowering the temperature, in our setup, we measured the response of the whole system. Here, the quartz resonator is effectively connected in series with a biasing circuit (∼1M R_4_ and R_5_ in parallel with a small ∼50 pF input capacitance of T_1_). This configuration may limit the apparent quality factor of the test circuit.

**Figure 3:**
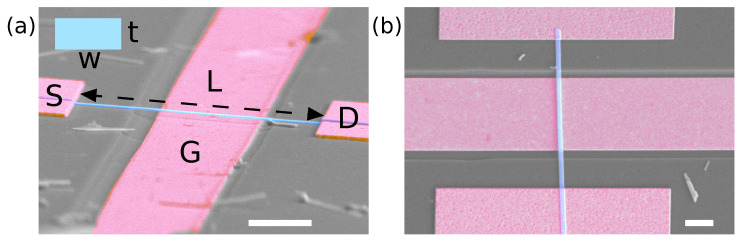
(**a**) Scanning electron microscopy images of the tested devices with source (S), drain (D) and gate (G) electrodes in an angled view (device No. 1) (**b**) and top view (device No. 2). Scale bar: 2 μm. A false colour was added for clarity, where magenta highlights the deposited source, drain and gate electrodes, whilst the suspended nanowire is highlighted in blue. The rectangular cross section of the Bi_2_Se_3_ nanowire is shown in the schematic inset with width *w* and thickness *t*.

Two Bi_2_Se_3_ nanowire devices with small and large thickness to length ratios (1:34 for device No. 1 and 1:237 for device No. 2) were selected to test the circuit and examine their resonant response (Figure 3). The relevant geometrical parameters of the nanowires, S-D resistance and measured and calculated resonant frequencies are shown in Table 1.

Figure 2b compares the amplitude spectra obtained for a Bi_2_Se_3_ nanowire device (No. 1) with a thickness of 224 nm and a length of 7.6 μm with a test circuit, where the nanowire was substituted with a similar resistance (20 kΩ) resistor. In contrast with the quartz resonator, the amplitude for both systems is negative. These differences can be understood by considering that the quartz resonator is connected between G and D terminals (Figure 1) and at resonance, its impedance becomes very low, raising the overall system gain to a positive ∼13 dB. However, for nanowires, the system detects a small modulation in the DC conductivity between S and D terminals of the nanowire induced by the gate electrode. As the transconductance of the gated nanowire is very low in comparison with, e.g., a regular field-effect transistor, the net gain of the system remains negative. At the same time, this does not hinder the detection of clear differences between the nanowire and the resistor spectra for frequencies up to approximately 2 MHz. Here, the spectrum with the nanowire shows a positive peak at approximately 1.20 MHz, followed by a negative peak at 1.52 MHz. We associate these antisymmetric peaks with the mechanical resonance of the nanowire. Near the resonance, the phase of the mechanical oscillations changes relative to the phase of the driving electric field from the gate electrode. The observed net signal is a sum of several factors, including capacitive coupling and cross-talk between the wires. The asymmetric shape of the gated nanowire spectra may be explained by constructive and destructive interference between the modulated nanowire signal and all other contributing factors. Similar pairs of positive–negative peaks associated with a phase change, as well as negative amplitudes of the resonance signal, have been reported in the literature [14,33,34,35]. Since the gate-induced electric field depends on the distance to the nanowire, the response may become nonlinear at large oscillation amplitudes. Another explanation of the asymmetric spectral peaks could be the positive and negative mechanical feedback due to in-phase and out-of-phase oscillations [36].

To calculate the expected resonant frequencies, we use the dynamic Euler–Bernoulli equation for the fundamental resonant frequency $f_{0}$ of a double-clamped beam [37]:

$$f_{0}=\frac{22.4}{2\pi L^{2}}\sqrt{\frac{EI}{\rho A}},$$

where *L*—suspended length, *E*—Young’s modulus, *I*—area moment of inertia, ρ—density and *A*—actuation area. For *E*, the room temperature value of 44 GPa, determined for Bi_2_Se_3_ nanoribbons via the electromechanical resonance method [9], was used. Whilst the most interesting quantum phenomena of Bi_2_Se_3_ occur at low temperatures, there is currently very little experimental data about the elastic and thermoelastic properties of nanoscale Bi_2_Se_3_ at low temperatures to the best of our knowledge. A recent study has reported the resonance of two double-clamped Bi_2_Se_3_ nanowire devices [12] at millikelvin temperatures. In this report, for a device with nanowire dimensions of a width of 105 nm, a thickness of 116 nm and a length of 1.5 μm, a resonant frequency of 115 MHz was determined, which is 10% lower than estimated using a room temperature Young’s modulus value of 44 GPa [9]. It must be taken into account that nanomaterials may exhibit size dependence in the Young’s modulus and its temperature dependence may differ from its bulk counterpart [38,39]. However, for Bi_2_Se_3_ nanoribbons in particular, in a thickness range of 30 to 100 nm, no size dependence has been reported [9].

By comparing the results for device No. 1 and device No. 2, it is apparent that the device with the thickest nanowire (No. 1) exhibits an almost 7 times lower frequency than expected, while the thinner nanowire (No. 2) matches closely with the expected value. The discrepancies in the expected frequency could be explained by changes in clamping of the nanowire ends due to differential expansion or compression of the nanowire and its contact with material during cooling and heating cycles.

**Figure 4:**
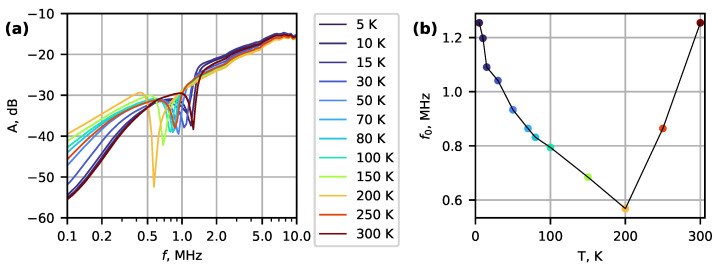
(**a**) Amplitude spectra of a Bi_2_Se_3_ nanowire (device No. 2) at different temperatures from 300 K to 5 K. (**b**) Negative peak frequency $f_{0}$ dependence on temperature.

For the thin nanowire device (No. 2), a series of spectra were recorded while heating the device from 5 K to 300 K (Figure 4). The resonant frequency decreased almost two-fold with increasing temperature. Then, it reached a minimum value at about 200 K and increased back to its low-temperature value. The observed temperature response is significant and can be compared to a graphene monolayer resonator [40,41], which exhibits an up to two-fold increase in frequency while cooling from 300 K to 125 K. However, for graphene, the upwards shift in frequency came from the isotropic contraction of metal contacts that were also suspended. In contrast, for double- and single-clamped Si [15] and GaN [42] nanowires, a frequency increase of only up to 1% has been observed down to 12 K, attributed to a temperature-dependent Young’s modulus and thermal contraction of the resonating materials.

The unexpected upwards shift in the resonant frequency from 200 K to 300 K was observed in repeated measurements for this sample and requires further investigation to determine if its origin is connected to differential thermal compression/expansion of the structure leading to changes in the boundary conditions of the resonator.

**Figure 5:**
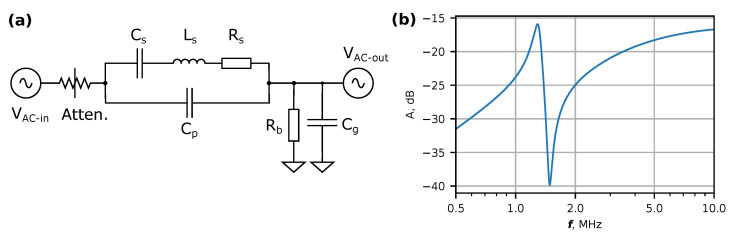
(**a**) Equivalent circuit diagram of the Bi_2_Se_3_ nanowire resonator. Element values: C_s_—0.3 pF; L_s_—50 mH; R_s_—20 kΩ; C_p_—1 pF; R_b_—20 kΩ; C_g_—1 pF; 10 dB attenuator (Atten.) models the coupling strength of the gate electrode. (**b**) Simulated response of (**a**) with aforementioned parameters shows asymmetric shape of the resonance spectrum, qualitatively similar to that of device No. 1.

The differences in observed line shapes for both samples could be accounted for by different geometries and *Q* factors that all contribute to phase shifts in resonant systems. To understand the occurrence of asymmetric line shapes and their different appearance for both examined devices, the expected response was simulated using an equivalent circuit (Figure 5a), which is typical for modeling NEM resonators [43]. Without any other components, the network of C_s_, L_s_ and R_s_ would produce a series resonance that can be observed as a positive peak in the amplitude transfer function. With C_p_ added parallel to the series network, a negative peak is introduced in the spectrum. Such pairs of positive and negative peaks in experimentally measured NEMS transfer functions have been reported previously [14,43]. In our equivalent circuit, we included an additional resistor R_b_ and capacitor C_g_, which represent the biasing resistors in a real circuit and the effective input capacitance of the MOSFET amplifier. In the presence of R_b_ and C_g_, the positive peak broadens and reduces in magnitude in comparison to the negative peak (Figure 5b). The input attenuator Γ characterizes the coupling strength of the gate electrode and the nanowire. The simulated spectrum of $A=20\log_{10}\left|\frac{V_{ac-out}}{V_{ac-in}}\right|-\Gamma$ (Figure 5b) with element values detailed in the figure caption corresponds well with measured spectrum of device No. 1 (Figure 2b). The choice of element value combinations in the equivalent circuit changes the relative magnitude of the positive and negative peaks and can make the positive peak less prominent as in the case of device No. 2.

**Figure 6:**
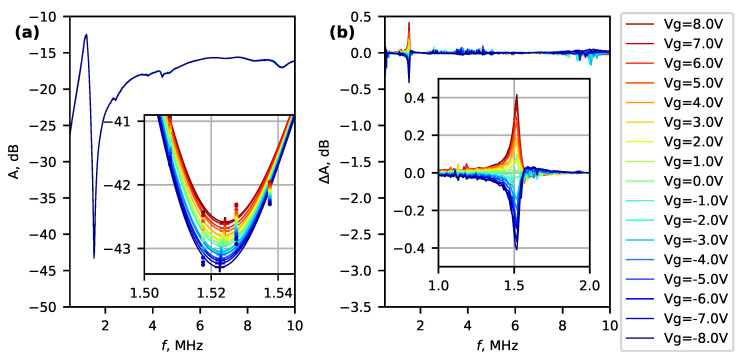
(**a**) Amplitude spectra of system with a Bi_2_Se_3_ nanowire (device No. 1) and a preamplifier at different gate voltages at 8 K temperature. (**b**) Difference in the amplitude spectra from the mean spectrum. Insets show a narrow region of spectra near 1.5 MHz.

For Bi_2_Se_3_ nanowire device No. 1, a series of spectra $A\left(f\right)=|V_{AC-out}\left(f\right)|/|V_{AC-in}\left(f\right)|$ were recorded at 8 K at different bias voltages $V_{g}$ (Figure 6a) to investigate the frequency and amplitude dependence on the gate voltage.

Small systematic changes were detected by comparing spectra at different gate voltages. Figure 6b shows the deviation ΔA from the mean spectrum recorded at $V_{g}$ intervals from −8 V to +8 V. The maximum difference occurs at approximately 1.52 MHz, which corresponds to the minimum of the original spectra (Figure 6a). In order to find the amplitude and frequency changes, the spectra near 1.52 MHz were fitted with a polynomial function. The extreme points of the fitted polynomial are marked by “+” in the inset of Figure 6a.

**Figure 7:**
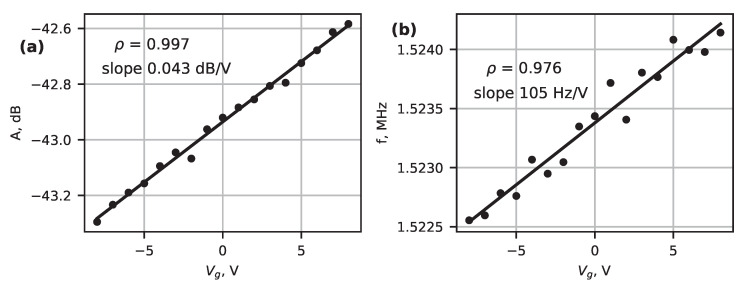
Extreme amplitude (**a**) and frequency (**b**) dependence on gate voltage for device No. 1.

Figure 7 shows the linear amplitude and frequency response to the applied gate voltage, which decreases for negative voltage values and increases for positive values. For the nanowire dimensions characterized in our study, we can use the continuum model to describe the force that is exerted by AC and DC voltages by

$$F_{ext}\left(x,t\right)=\frac{1}{2}\frac{dc_{g}}{du}\left(x\right)\left(V_{g}^{2}+\frac{1}{2}V_{g}^{ac^{2}}+2V_{g}V_{g}^{ac}cos\left(2\pi ft\right)+\frac{1}{2}V_{g}^{ac^{2}}cos\left(4\pi ft\right)\right),$$

where $c_{g}$—capacitance per unit length and u(x)—displacement. Assuming the AC voltage is much smaller than the DC voltage, the quadratic terms in the equation can be neglected. A parabolic dependence on $V_{g}$ is expected; however, the relation observed in this study may appear linear due to the low-tunability of the device.

The anti-symmetric amplitude and frequency dependence around zero $V_{g}$ can be explained by work function differences between the Bi_2_Se_3_ and Au electrode materials, similar to [17]. The changes in amplitude (Figure 7a) and frequency (Figure 7b) were relatively small, at 0.043 dB/V and 105 Hz/V, in comparison to, e.g., graphene modulation in [29], which was in the MHz/V range. One possible reason for the low tunabilty could be the thickness of the nanowire. For example, in a 2D layered ReS_2_ nanoresonator device [44], a device with a thickness of 106 nm exhibited no tunability, while a 7 nm-thick device exhibited pronounced gate-induced frequency changes. The mechanical strain induced in the nanowire during cooling can also reduce the gate effect.

Nevertheless, there is a strong correlation between amplitude and frequency and the applied DC voltage, confirming the tunability of the system. Other features in the spectra did not exhibit as high a correlation, supporting the conclusion that the chosen frequency represents the mechanical resonant frequency of the nanowire.

## 4. Conclusions

We have presented an electrical preamplifier circuit for the detection of 1D nanomaterial resonance inside a cryostat. Placing the preamplifier in close vicinity of the resonator allows minimization of the contribution from the parasitic wire capacitance and enables facile readouts at the same frequency as the excitation. The temperature and gate voltage dependence of the resonant frequency was investigated for Bi_2_Se_3_ nanowire samples at temperatures as low as 5 K, showing large changes in resonant frequency with temperature and the possibility to tune the oscillations with gating. Samples with higher transconductances possibly using thinner Bi_2_Se_3_ nanowires would allow obtaining a higher response from the system to understand in detail how the elastic properties change with temperature. This would allow a precise NEM switch design for operation at low temperatures for future quantum and space technologies. Although the system was demonstrated using Bi_2_Se_3_ nanowires, the circuit is a generic common drain amplifier and may be used for studies of other NEMSs at low temperatures.

## Figures and Tables

**Table 1:** Geometrical parameters (length *L*, thickness *t*, width *w*), resistance *R*, measured $f_{meas}$ and calculated $f_{calc}$ resonant frequencies of the Bi_2_Se_3_ resonators.

Device	L,μm	*t*, nm	*w*, nm	R,kΩ	$f_{\mathrm{meas}}$, MHz	$f_{\mathrm{calc}}$, MHz
No. 1	7.6	224	124	50	1.5	9.8
No. 2	8.3	35	266	36	1.2	1.2

## Data Availability

The data presented in this study are available on request from the corresponding authors.
